# Triggers, clinical spectra and outcomes of pediatric anaphylaxis in a tertiary center: impact of comorbidities and cofactors on severity

**DOI:** 10.1186/s13223-026-01033-1

**Published:** 2026-06-19

**Authors:** Necmiye Keser-Ozturk, Yasir Maghdeed, Selcen Bozkurt, Melek Yorgun-Altunbas, Salim Can, Razin Amirov, Ramin Mahmudov, Burkay Cagan Colak, Emel Eksi-Alp, Sevgi Bilgic-Eltan, Ahmet Ozen, Safa Baris, Elif Karakoc-Aydiner

**Affiliations:** 1 Division of Allergy and Immunology, Department of Pediatrics, Istanbul Jeffrey Model Diagnostic and Research Center for Primary Immunodeficiencies, The Isil Berat Barlan Center for Translational Medicine, Immune Deficiency Research and Application Center, Faculty of Medicine, Marmara University, European Academy of Allergy and Clinical Immunology Marmara University Hospital Center of Excellence, Istanbul, Turkey; 2 Department of Pediatrics, Faculty of Medicine, Marmara University, Istanbul, Turkey; 3 Division of Pediatric Emergency Medicine, Department of Pediatrics, Faculty of Medicine, Marmara University, Istanbul, Turkey

**Keywords:** Pediatric anaphylaxis, Triggers, Comorbidities, Cofactors, Severity, Biphasic reactions, Risk factors

## Abstract

**Purpose:**

Anaphylaxis is a potentially life-threatening systemic hypersensitivity reaction. While triggers, clinical manifestations, and severity are influenced by age and sociocultural factors, most evidence regarding the impact of comorbidities and cofactors comes from adult studies. This study aimed to characterize the triggers, clinical features, and outcomes of pediatric anaphylaxis in a tertiary care center, with a particular emphasis on risk factors for severity.

**Methods:**

We retrospectively reviewed records from August 2023–August 2024 at the European Allergy Academy and Clinical Immunology Center of Excellence in Istanbul. Children aged 0–18 years (*n* = 100) with anaphylaxis as defined by the 2020 World Allergy Organization (WAO) criteria were included. Demographic, clinical, and follow-up data were collected. The updated 2024 (WAO) grading system was used to classify severity.

**Results:**

Of the 3,959 records, 100 children fulfilled the inclusion criteria. A female predominance was noted in those ≤ 4 years, with males predominating thereafter. Medications were the leading trigger, followed by food, venom, and idiopathic causes. Foods were more prevalent among outpatients, and drugs were more prevalent among inpatients. Comorbidities were more common in hospital-onset anaphylaxis. Atopy was more common in food-induced anaphylaxis, whereas infections and polypharmacy were more common in drug-induced cases. Food-related reactions with endogenous factors were typically milder, whereas drug-related reactions—especially in older patients with polypharmacy or hospital-onset—tended to be more severe. Biphasic reactions were associated with delayed presentation, elevated WBC/ANC, and older age. Intramuscular adrenaline was administered in 95% of the patients, but prehospital autoinjector use was rare.

**Conclusion:**

Our findings underscore the need for early recognition and management, systematic evaluation of contributing factors, risk-adapted monitoring, and individualized follow-up in pediatric anaphylaxis patients.

## Introduction

Anaphylaxis is a potentially life-threatening systemic reaction, with a pediatric incidence ranging from 1 to 761 per 100,000 annually [1]. Its frequency and triggers vary with sociocultural, environmental, and geographic factors [2, 3]. The diagnosis is clinical, and intramuscular epinephrine remains the first-line treatment [4]. Underprescription of adrenaline autoinjectors (AAI), reluctance to self-administer, and delayed recognition and treatment in both primary and emergency care settings remain major barriers to the appropriate management of anaphylaxis [5, 6]. Given recurrence rates of up to 54%, postepisode follow-up in allergy and immunology clinics is essential for identifying triggers, implementing preventive strategies, and reducing morbidity and mortality [4, 7]. Although comorbidities and endogenous/exogenous factors can influence the risk and severity of anaphylaxis—with their impact potentially varying by age and trigger—most available evidence comes from adult studies, limiting their applicability to pediatric cases [8–13].

This study was designed to evaluate the triggers, comorbidities, potential factors, clinical manifestations, treatment approaches, and postdischarge follow-up characteristics of pediatric patients (0–18 years) diagnosed with anaphylaxis at the European Allergy Academy and Clinical Immunology Center of Excellence in Istanbul. In particular, this study aimed to investigate the influence of etiologic variations, coexisting medical conditions, and endogenous and exogenous factors on the risk and severity of anaphylaxis—an area that remains underexplored in the pediatric population. By addressing this gap in the literature, the present study seeks to contribute to a more comprehensive understanding of the clinical risk profiles of childhood anaphylaxis.

## Methods

### Study design and population

Systematic cross-sectional screening of hospital electronic medical records at a pediatric tertiary care center was conducted between August 2023 and August 2024 to identify patients aged 0–18 years who had undergone serum tryptase testing or had been prescribed an AAI. In addition, records from the pediatric allergy and immunology outpatient clinic were reviewed to identify patients with relevant ICD-10 diagnostic codes, including “Allergy, unspecified (T78.4), Anaphylactic shock, unspecified (T78.2), Anaphylactic shock due to adverse food reaction (T78.0), Other adverse food reactions, not elsewhere classified (T78.1), Idiopathic urticaria (L50.1), Urticaria, unspecified (L50.9), and Angioneurotic edema (T78.3)”.

### Patient identification and inclusion criteria

Medical records were independently reviewed by two investigators (Y.M. and N.K.O.) to identify confirmed cases of anaphylaxis. Repeated emergency department visits related to the same anaphylactic episode were consolidated into a single entry. The severity of reactions was assessed according to the World Allergy Organization (WAO) classification system [14]. According to this classification, grade 1–2 reactions were defined as nonanaphylactic systemic hypersensitivity reactions, whereas grade 3–5 reactions met the diagnostic criteria for anaphylaxis. Only patients with grade 3–5 reactions fulfilling the 2020 WAO anaphylaxis criteria were included in the study [4]. Patients with grade 1–2 systemic hypersensitivity reactions, incomplete and/or insufficient medical documentation, and misclassified or miscoded diagnoses were excluded. For data interpretation, the severity grading was further simplified by categorizing Grade 3 reactions as mild anaphylaxis and Grade 4–5 reactions as severe anaphylaxis.

### Data collection

The study dataset included demographic characteristics, identified triggers, clinical manifestations, physical examination findings, and vital signs recorded in the emergency department. Treatment details—including both prehospital and in-hospital interventions—were collected alongside comorbidities, endogenous and exogenous cofactors, history of previous allergic reactions, and family history of atopy. The laboratory data included admission and baseline serum tryptase levels, complete blood count parameters, total and allergen-specific IgE levels, and skin prick test results. Postdischarge management, including allergen avoidance recommendations, AAI prescription, provision of an anaphylaxis action plan (AAP), and allergy clinic follow-up, was also documented. All patient data were recorded via a standardized data collection form.

Triggers of anaphylaxis were primarily identified based on documented patient history. In patients with post-event follow-up in the pediatric allergy and immunology clinic, skin prick testing and/or serum-specific IgE measurements were assessed to support trigger identification.

Biphasic reactions were defined as the recurrence of anaphylactic symptoms after complete resolution of the initial episode, in the absence of re-exposure to the triggering allergen, occurring within 72 h.

### Ethical approval

Ethical approval was obtained from the Ethics Committee of Marmara University, Faculty of Medicine (Approval number/date: 09.2024.1085/16.10.2024).

### Statistical analysis

All the statistical analyses were performed via Jamovi software (version 2.3.28). Descriptive statistics were used to summarize the study population. Continuous variables were expressed as the means with standard deviations (SDs) or medians with interquartile ranges (IQRs), whereas categorical variables were reported as frequencies and percentages. Categorical variables were compared via Pearson’s chi-square test or Fisher’s exact test, as appropriate. For continuous variables, Student’s *t* test or the Mann–Whitney *U* test was applied according to the data distribution. Logistic regression analysis was performed to assess the potential impact of cofactors on the occurrence of anaphylaxis. A two-tailed *p* value of < 0.05 was considered statistically significant.

## Results

### Study population

**Fig. 1:**
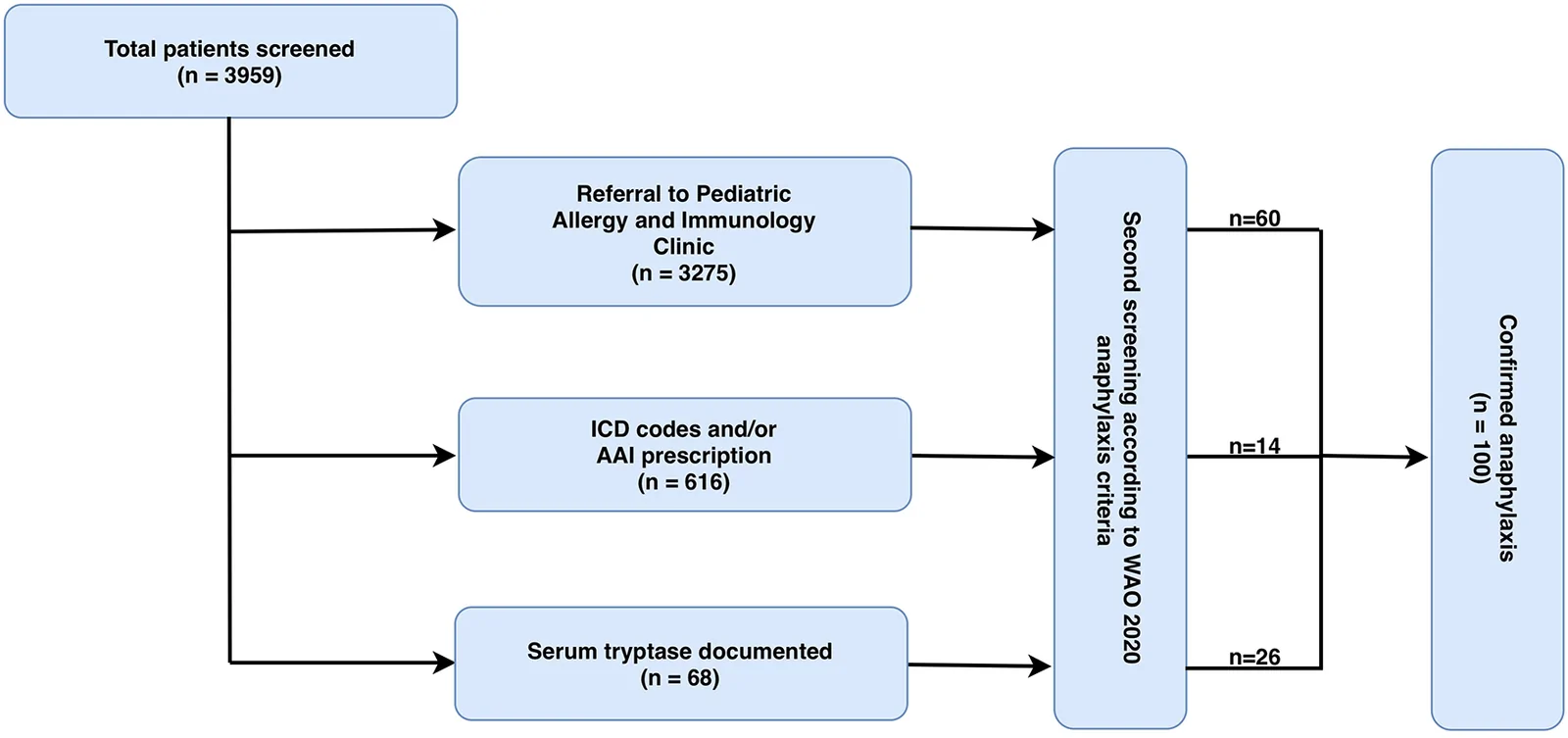
Flow diagram of screening and eligibility assessment for pediatric anaphylaxis patients. The data are presented as absolute numbers. *n*, number

Between August 2023 and August 2024, patient records were identified from three sources: the Pediatric Allergy and Immunology Clinic (*n* = 3,275), ICD code screening and/or adrenaline auto-injector (AAI) prescriptions (*n* = 616), and documented serum tryptase measurements (*n* = 68), yielding a total of 3,959 records. All records were screened independently by two investigators (Y.M. and N.K.O.). Following secondary review, 100 patients fulfilling the WAO diagnostic criteria for anaphylaxis were included in the study [4] (Fig. 1).

### Demographic and clinical characteristics

The demographic, clinical, and therapeutic characteristics of the study cohort are summarized in Table 1. An age-dependent difference in sex distribution was observed: in children aged ≤ 4 years, anaphylaxis was more common in females (61.5%) than in males (38.5%); in those > 4 years, males predominated (62% vs. 38%) (*p* = 0.036). Cardiovascular involvement was significantly more common in patients older than 12 years (*p* = 0.002), whereas no significant difference was noted between patients aged ≤ 4 years and those > 4 years.

**Table 1:** Demographics, clinical features, treatments, and outcomes in pediatric anaphylaxis

	*n* = 100 (100%)
*Sex*	
Male, *n* (%)	56 (56%)
Female, *n* (%)	44 (44%)
*†Age (years), median (IQR; 25–75)*	7.5 (3.9–13.8)
≤ 4 years, *n* (%)	26 (26%)
> 4 years, *n* (%)	74 (74%)
*Past medical history for allergies*	
History of previous anaphylaxis, *n* (%)	19 (19%)
History of type 1 reaction with the same trigger, *n* (%)	12 (12%)
Previous follow-up in an allergy outpatient clinic, *n* (%)	24 (24%)
Possession of AAI before the visit, *n* (%)	7 (7%)
Use of AAI before the visit, *n* (%)	1(1%)
*Atopy, n (%)*	47 (47%)
Food allergy, *n* (%)	20 (20%)
Drug allergy, *n* (%)	7 (7%)
Venom allergy, *n* (%)	3 (3%)
Allergic rhinitis, *n* (%)	12 (12%)
Asthma, *n* (%)	17 (17%)
Atopic dermatitis, *n* (%)	9 (9%)
‡ Other	7 (7%)
*Family history of atopy, n (%)*	10 (29%)
*Reaction site, n (%)*	100 (100%)
Inpatient, *n* (%)	44 (44%)
Outside the hospital, *n* (%)	56 (56%)
Self-admission, *n* (%)	46 (82%)
Via ambulance, *n* (%)	10 (18%)
*†Time elapsed till emergency department arrival (minutes), median (IQR; 25–75)*	42.5 (28.0–60.0)
*†Laboratory features*	
Triptase level in the emergency admission (ug/L), median (IQR; 25–75)	9.5 (6.8–13.0)
*Baseline triptase level (ug/L), median (IQR; 25–75)	4.6 (3.8–6.0)
Total IgE (IU/ml), median (IQR; 25–75)	142 (30–354)
Absolute eosinofil count, median (IQR; 25–75)	125 (20–300)
*Treatment, n (%)*	
Adrenalin, *n* (%)	95 (95%)
Antihistamine, *n* (%)	77 (77%)
Methylprednisolone, *n* (%)	69 (69%)
IV bolus fluid, *n* (%)	20 (20%)
Bronchodilator, *n* (%)	14 (14%)
Mechanical ventilation	1 (1%)
Others	2 (2%)
*Outcome*	
Frequency of biphasic reactions	14 (14%)
†Time elapsed after the initial reaction (hours), median (IQR; 25–75)	5.0 (1.5–8.0)
†Observation time in the emergency department (hours) median (IQR; 25–75)	24.0 (24.0–24.0)
Admission to PICU, *n* (%)	5 (5%)
Fatal reaction, *n* (%)	0 (0%)
AAI at discharge, *n* (%)	41 (41%)
AAP at discharge, *n* (%)	76 (76%)
Postdischarge pediatric allergy clinic follow-up, *n* (%)	50 (50%)
†Postdischarge pediatric allergy clinic follow-up (days), median (IQR; 25–75)	31 (9–40)

^†^Categorical variables were presented as *n* (%). Nonparametric continuous variables were presented as median (IQR, 25–75%). ‡ Other: Chlorine allergy, urticaria after swimming (*n* = 1), aeroallergen sensitivity (*n* = 5), chronic urticaria and allergic rhino-conjunctivitis (*n* = 1). *Among the cohort, one patient had pre-existing cutaneous mastocytosis; three others had baseline tryptase > 8 ng/mL and are undergoing evaluation for possible mastocytosis

AAI: adrenaline autoinjector; AAP: Anaphylaxis action plan; IgE: Immunglobulin E; IQR: Interquartile Range; IV: intravenous; PICU: Pediatric intensive care unit; *n*: number

### Comorbidities, factors and cofactors influencing anaphylaxis

**Fig. 2:**
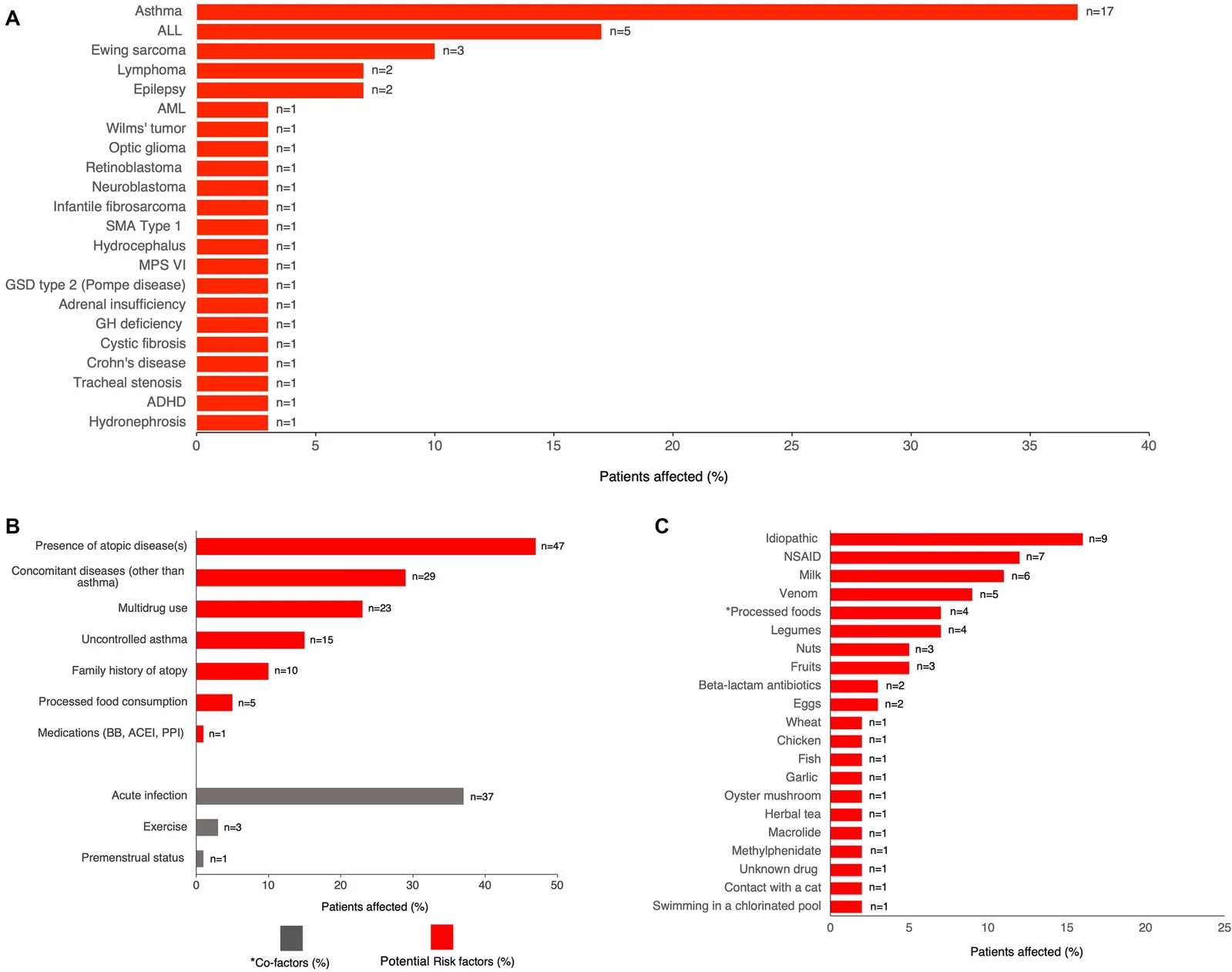

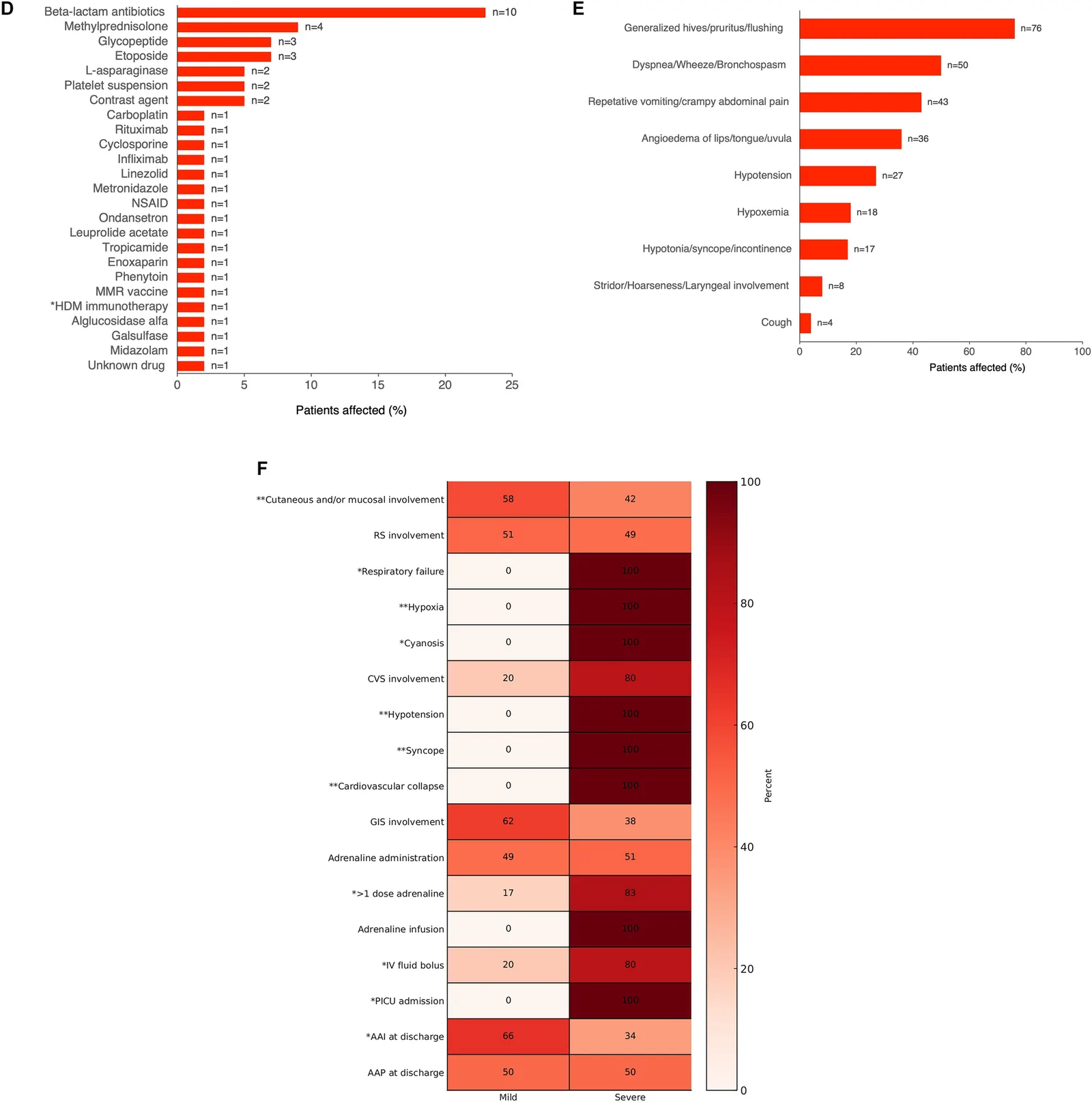
Key epidemiological, clinical, and management features of pediatric anaphylaxis. **A** Spectrum of comorbidities observed in the study cohort. **B** Cofactors and Potential Risk Factors in Anaphylaxis. **C** Triggers of community-onset anaphylaxis. *Processed foods: packaged, commercially prepared foods containing additives. **D** Triggers of anaphylaxis in hospitalized patients. *HDM: house dust immunotherapy. **E** Clinical presentation of anaphylaxis. **F** Distribution of clinical features and management according to severity [mild (grade 3) vs. severe (grades 4–5)]. The heatmap displays percentages, with darker colors indicating higher values and lighter colors indicating lower values. Categorical variables were compared via the χ^2^ test or Fisher’s exact test, and *p* < 0.05 was considered statistically significant. *Denotes variables with p < *0.05;* **Denotes variables with p < *0.001.* In panels **A**–**E**, the data are presented as numbers and percentages. *n*, number. ALL, acute lymphoblastic leukemia; AML, acute myeloid leukemia; SMA, spinal muscular atrophy; MPS, mucopolysaccharidosis; GSD, glycogen storage disease; GH, growth hormone; ADHD, attention-deficit/hyperactivity disorder; BB, beta-blocker; ACEI, angiotensin-converting enzyme inhibitor; PPI, proton pump inhibitor; NSAID, nonsteroidal anti-inflammatory drug

Comorbidities were identified in 46 patients. Asthma was the most frequently reported comorbidity (37%, *n* = 17), followed by malignancy (35%, *n* = 16). The full spectrum of comorbid conditions is presented in Fig. 2a. Comorbidities were significantly more prevalent among patients who developed anaphylaxis during hospitalization than among those with community-onset reactions [70% vs. 30%, *p* < 0.001].

Factors and cofactors influencing anaphylaxis were systematically assessed (Fig. 2b). Thirteen patients (13%) had no identifiable factors; the remainder had 1–4 factors, with a median of 1 (IQR: 1–2) per patient. Exercise-induced anaphylaxis was observed in three patients. In two of these cases, wheat (*n* = 1) and walnut (*n* = 1) were identified as causative allergens. One patient with a prior history of urticaria triggered by chlorinated water developed anaphylaxis following swimming in a chlorinated pool.

### Triggers of anaphylaxis

Medications were the most common overall trigger (*n* = 52), followed by food (*n* = 28), insect venom (*n* = 5), other identifiable causes (*n* = 6), and idiopathic cases (*n* = 9). Among outpatients, food was the leading cause (*n* = 28), followed by medications (*n* = 12), idiopathic reactions (*n* = 9), insect venom (*n* = 5), and other exposures (*n* = 2) (Fig. 2c). In hospitalized patients, drugs were the predominant trigger (*n* = 40); other less common triggers included platelet transfusion (*n* = 2), house dust mite immunotherapy (*n* = 1), and diphtheria–tetanus–pertussis (DTP) vaccination (*n* = 1) (Fig. 2d).

### Clinical manifestations and laboratory findings

The distribution of clinical manifestations observed in our cohort is summarized in Fig. 2e. Cutaneous symptoms were the most common, followed by respiratory, gastrointestinal, and cardiovascular involvement. Tryptase levels were measured during the acute phase in 52 patients, and baseline levels were obtained in 24 patients; median values are presented in Table 1. Respiratory system involvement was significantly associated with baseline tryptase levels (*p* = 0.044) but not with acute-phase tryptase levels (*p* = 0.89).

### Comparative analyses

Comparative data on food-induced (FIA) vs. non–food-induced (NFIA) and drug-induced (DIA) vs. non–drug-induced anaphylaxis (NDIA) are summarized in Table 2. Atopy was significantly more common in patients with FIA than in those with NFIA (*p* < 0.001; OR: 6.9, 95% CI 2.47–19.2; RR: 2.26). Among exogenous cofactors, concurrent infection was more frequently observed in DIA than in NDIA (*p* = 0.017; OR: 2.78, 95% CI 1.19–6.5; RR: 1.92). Polypharmacy was strongly associated with DIA (*p* < 0.001; OR: 15.6, 95% CI 3.4–71.2; RR: 9.69). Additionally, DIA was significantly more likely to occur in hospital settings (*p* < 0.001; OR: 36.6, 95% CI 11–123; RR: 9.23).

**Table 2:** Comparison of food or drug-induced versus non-induced anaphylaxis

		Food-induced Anaphylaxis	Non-food anaphylaxis		Drug-induced anaphylaxis	Non-drug anaphylaxis	
		% (*n*)	% (*n*)	*p* value	% (*n*)	% (n)	*p* value
Sex	Female	23 (n = 10)	77(n = 34)	0.293	45 (n = 25)	55 (n = 31)	0.098
	Male	32 (n = 18)	68(n = 38)		61 (n = 27)	39 (n = 17)	
Age	≤ 4 years	46 (n = 12)	54 (n = 14)	**0.017**	50 (n = 13)	50 (n = 13)	0.812
	> 4 years	22 (n = 16)	78 (n = 58)		53 (n = 39)	47 (n = 35)	
Prior anaphylaxis	+	47 (n = 9)	53 (n = 10)	**0.037**	47 (n = 7)	53 (n = 12)	0.144
	−	23 (n = 19)	77 (n = 62)		56 (n = 45)	44 (n = 36)	
Prior reaction to same trigger	+	42 (n = 5)	58 (n = 7)	0.301	17 (n = 2)	83 (n = 10)	**0.009**
	−	26 (n = 23)	74 (n = 65)		57 (n = 50)	43 (n = 38)	
Prior allergy follow-up	+	50 (n = 12)	50 (n = 12)	**0.006**	25 (n = 6)	75 (n = 18)	**0.002**
	−	21 (n = 16)	79 (n = 60)		60 (n = 46)	40 (n = 30)	
Comorbidity	+	17 (n = 8)	83 (n = 38)	**0.029**	76 (n = 35)	24 (n = 11)	** < 0.001**
	−	37 (n = 20)	63 (n = 34)		31 (n = 17)	68 (n = 37)	
Atopy	+	47 (n = 22)	53 (n = 25)	** < 0.001**	32 (n = 15)	68 (n = 32)	** < 0.001**
	−	11 (n = 6)	89 (n = 47)		70 (n = 37)	30 (n = 16)	
Family history of atopy	+	50 (n = 5)	50 (n = 5)	0.272	10 (n = 1)	90 (n = 9)	0.051
	−	29 (n = 7)	71 (n = 17)		57 (n = 51)	43 (n = 39)	
Skin involvement	+	32 (n = 27)	68 (n = 57)	**0.036**	47 (n = 40)	53 (n = 44)	**0.047**
	−	6 (n = 1)	94 (n = 15)		75 (n = 12)	25 (n = 4)	
RS involvement	+	26 (n = 18)	74 (n = 52)	0.434	53 (n = 37)	47 (n = 33)	0.791
	−	33 (n = 10)	67 (n = 20)		50 (n = 15)	50 (n = 15)	
CVS involvement	+	15 (n = 6)	85 (n = 34)	**0.018**	65 (n = 26)	35 (n = 14)	**0.042**
	−	37 (n = 22)	63 (n = 38)		43 (n = 26)	57 (n = 34)	
GIS involvement	+	33 (n = 14)	67 (n = 28)	0.312	41 (n = 17)	59 (n = 25)	0.052
	−	15 (n = 8)	85 (n = 44)		60 (n = 35)	40 (n = 23)	
Anaphylaxis grade	Grade 3	43 (n = 22)	57 (n = 29)	** < 0.001**	35 (n = 18)	65 (n = 33)	** < 0.001**
	Grade 4–5	12 (n = 6)	88 (n = 43)		69 (n = 34)	31 (n = 15)	
PICU admission	+	0 (n = 0)	100 (n = 5)	0.319	100 (n = 5)	0 (n = 0)	0.057
	−	30 (n = 28)	70 (n = 67)		49 (n = 47)	51 (n = 48)	
AAI at discharge	+	51 (n = 21)	49 (n = 20)	** < 0.001**	12 (n = 5)	88 (n = 36)	** < 0.001**
	−	12 (n = 7)	88 (n = 52)		80 (n = 47)	20 (n = 12)	
AAP at discharge	+	28 (n = 21)	72 (n = 55)	0.881	47 (n = 36)	53 (n = 40)	0.094
	−	29 (n = 7)	71(n = 17)		67 (n = 16)	33 (n = 8)	
Allergy follow-up	+	44 (n = 22)	56 (n = 28)	** < 0.001**	28 (n = 14)	72 (n = 36)	** < 0.001**
	−	12 (n = 6)	88 (n = 44)		76 (n = 38)	24 (n = 12)	

Statistical comparisons were performed using the χ² or Fisher’s exact test for categorical variables. Bold values indicate variables that are statistically significant (p < 0.05). RS, Respiratory System; CVS, Cardiovascular System; GIS, Gastrointestinal System; PICU, Pediatric Intensive Care Unit; AAI, Adrenaline Auto-Injector; AAP, Anaphylaxis Action Plan.

### Severity of anaphylaxis

A comparison of mild versus severe anaphylaxis in relation to potential contributing factors is summarized in Table 3, and additional factors significantly associated with severity are illustrated in Fig. 2f. Age ≥ 12 years (*p* = 0.021) and in-hospital onset (*p* = 0.009) were significantly associated with greater anaphylaxis severity.

**Table 3:** Comparison of mild and severe anaphylaxis

		Mild anaphylaxis (Grade 3)	Severe anaphylaxis (Grade 4–5)	*p* value
		% (n)	% (n)	
Sex	Female	45 (n = 23)	43 (n = 21)	0.821
	Male	55 (n = 28)	57 (n = 28)	
Age	≤ 4 years	29 (n = 15)	22 (n = 11)	0.427
	> 4 years	71 (n = 36)	78 (n = 38)	
Prior anaphylaxis	+	25 (n = 13)	12 (n = 6)	0.091
	−	75 (n = 38)	88 (n = 43)	
Previous type 1 reaction with same trigger	+	14 (n = 7)	10 (n = 5)	0.586
	−	86 (n = 44)	90 (n = 44)	
Reaction setting	Inpatient Community	31 (n = 16)	57 (n = 28)	**0.015**
		69 (n = 35)	43 (n = 21)	
Mode of ED presentation	Self-referral Ambulance	89 (n = 32)	78 (n = 21)	0.303
		11 (n = 4)	22 (n = 6)	
Comorbidity	+	37 (n = 19)	55 (n = 27)	0.073
	−	63 (n = 32)	45 (n = 22)	
Presence of anaphylaxis influencing factors	+	88 (n = 45)	86 (n = 42)	0.708
	−	12 (n = 6)	14 (n = 7)	
Number of anaphylaxis influencing factors	1 or 2	90 (n = 46)	84 (n = 41)	0.332
	3 or 4	10 (n = 5)	16 (n = 8)	
Atopy	+	55 (n = 28)	37 (n = 19)	0.106
	−	45 (n = 23)	63 (n = 30)	
Uncontrolled asthma	+	14 (n = 7)	16 (n = 8)	0.716
	−	86 (n = 44)	84 (n = 41)	
Family History of Atopy	+	35 (n = 8)	18 (n = 2)	0.437
	−	65 (n = 15)	82 (n = 9)	
Infection	+	31 (n = 16)	43 (n = 21)	0.234
	−	69 (n = 35)	57 (n = 28)	
Multidrug usage	+	14 (n = 7)	32 (n = 16)	**0.025**
	−	86 (n = 44)	68 (n = 33)	

Statistical comparisons were performed using the χ^2^ or Fisher’s exact test for categorical variables. ED, emergency department.

Bold values indicate variables that are statistically significant (p < 0.05).

### Biphasic reactions

Biphasic reactions were significantly associated with longer intervals between symptom onset and emergency department presentation (*p* = 0.043), higher WBC counts (*p* = 0.023), elevated ANC (*p* = 0.015), and the administered adrenaline dose (*p* < 0.001). No significant associations were found between biphasic reactions and the duration of emergency department observation or baseline/acute-phase tryptase levels (*p* > 0.05). A moderate to strong positive correlation was identified between age and the time interval between the initial and biphasic reactions (Spearman’s rho = 0.68, *p* = 0.01).

### Postdischarge evaluation

After discharge, 50 patients were evaluated in the pediatric allergy and immunology clinic. Allergen-specific testing was performed via both skin prick testing (SPT) and serum-specific IgE in 11 patients, serum-specific IgE alone in 26 patients, and SPT only in 7 patients. The causative allergen was identified in 23 patients based on specific IgE and/or skin prick testing. Oral food challenges were not performed in 21 patients because test values were markedly above age-adjusted cut-off levels and all had a physician-diagnosed history of anaphylaxis. Diagnostic oral food challenges were planned for the remaining two patients, but their families declined.

Among the five patients with insect sting–related anaphylaxis, four were evaluated after discharge, confirming sensitization to Hymenoptera venom in all patients (three to honeybees and one to hornets, with specific IgE levels ≥ 0.35 kU/L). Among the nine patients initially diagnosed with idiopathic anaphylaxis, four underwent postdischarge evaluation, but no allergens were identified; the remaining five did not attend follow-up.

## Discussion

Anaphylaxis is the most severe form of systemic hypersensitivity reaction, and its etiology, clinical presentation, severity, and associated comorbidities and cofactors are influenced by age, sex, and regional factors [2, 4, 5, 15–17]. This study provides novel insights into pediatric anaphylaxis by systematically evaluating triggers, comorbidities, and cofactors in a tertiary care cohort using the 2020 WAO diagnostic criteria and the updated 2024 WAO grading system.

In line with previous reports [13, 15–18], anaphylaxis was more common in males overall, although females predominated in the ≤ 4-year group, and drug-induced reactions were more common among older and hospitalized patients, mainly related to β-lactam antibiotics in inpatients and NSAIDs in outpatients. Unlike most pediatric series from Europe, Asia and the United States, where food is consistently the leading trigger [3, 15, 19–21], we observed a predominance of drug-induced reactions. This likely reflects the high proportion of hospitalized patients in our cohort, highlighting regional and contextual differences in anaphylaxis etiology. Consistent with national data, food-induced reactions predominated in younger children and in the outpatient setting [2, 3], with a notable contribution from processed foods, underscoring the emerging concern of hidden allergens in pediatric cases [13, 18]. Unlike prior studies highlighting gastrointestinal symptoms in food-induced anaphylaxis [2, 3], our data indicated a predominance of cutaneous and mucosal symptoms. Rare healthcare-related triggers, including topical tropicamide, house dust mite immunotherapy, and DTP vaccination, as well as unusual outpatient triggers, such as cat contact in a sensitized patient—an association not previously documented—and chlorinated pool exposure were identified. Cardiovascular involvement was the least frequent manifestation but, similar to previous studies, was significantly associated with drug-induced reactions and age ≥ 12 years [2, 3, 19, 22–24]. Intramuscular adrenaline use was exceptionally high, surpassing previously reported pediatric rates [3, 19, 25–27], whereas prehospital AAI use remained low, which is consistent with global data [3, 26, 27]. Biphasic reactions occurred in 14% of our cohort—which is higher than previously reported rates [2, 3, 16, 19]—likely because of the longer observation period in our hospital. Biphasic events were associated with delayed presentation, higher WBC counts, and ANC values, suggesting that delayed care and systemic inflammation markers may predict biphasic risk. A strong positive correlation was identified between age and the interval to biphasic reactions (rho = 0.68, *p* = 0.01), suggesting that older children may require extended monitoring. Whereas most pediatric studies report Emergency Department (ED) stays of 4–6.5 h [2, 27, 28], current guidelines recommend risk-stratified observation after anaphylaxis, with a minimum monitoring period of 6–8 h in patients with respiratory involvement and 12–24 h in those with associated hypotension [29]. In this context, our findings indicate that a longer observation period may need to be considered in selected patient groups, particularly adolescents, those with delayed presentation, and those with elevated inflammatory markers. Several factors increase the risk of severe or fatal anaphylaxis, including advanced age and associated comorbidities, drug- or venom-induced reactions, and the need for hospital admission or prolonged observation [3, 12, 19, 30–32]. Data from the Polish Anaphylaxis Registry revealed that patients with a greater comorbidity burden had an increased risk of anaphylaxis, whereas physical exertion, alcohol, and lipid transfer protein (LTP) sensitization were identified as key cofactors that increased severity. However, these findings were derived from adult populations, as pediatric cases were not evaluated in this study [11]. In the Philippines, severe cases were strongly associated with comorbidities—particularly malignancy—and acute infections, with multivariate analysis identifying age and cofactors as the main predictors of severity [8].

In our cohort, FIA was associated with milder reactions, whereas drug-induced reactions were linked to greater severity. Severe FIA is often linked to high-risk allergens such as peanuts, tree nuts, fish, and shellfish [33], which were rare in our cohort, possibly explaining the lower severity observed. Older age (≥ 12 years) and in-hospital onset were associated with greater severity, and severity showed a weak but significant positive correlation with age. Various endogenous factors (age, sex, comorbidities, atopy, baseline tryptase, prior reactions) and exogenous factors (exercise, infections, alcohol, medications, stress) can influence the onset and severity of anaphylaxis [8, 9, 30, 34, 35]. In our cohort, unlike previous reports suggesting a protective effect of atopy against severe anaphylaxis [19, 30], we found no direct association between atopic history and severity. Similarly, the baseline/admission tryptase level, number of cofactors and potential risk factors, and onset-to-ED interval were not associated with severity, suggesting that tryptase is a useful diagnostic biomarker but has limited predictive value for pediatric severity. A weak inverse correlation between the absolute lymphocyte count and severity likely reflected malignancy/chemotherapy-related lymphopenia and severe reactions in this group. Polypharmacy had emerged as a potential risk factor for drug-induced anaphylaxis and was associated with increased severity. Although endogenous and exogenous factors are recognized as important modifiers of anaphylaxis risk [11, 18, 36, 37], their overall burden showed no clear association with reaction severity, highlighting the multifactorial and heterogeneous nature of pediatric anaphylaxis. Factors influencing anaphylaxis also affected the reaction patterns. Atopy was associated with food-induced anaphylaxis, whereas acute infections were linked to drug-induced reactions. This differs from prior reports emphasizing the role of infections in food-induced reactions or during allergen immunotherapy [37], and our findings suggest their potential involvement in drug-induced reactions. Notably, one patient developed exercise-induced anaphylaxis while swimming in a chlorinated pool, with a prior history of chlorine-induced urticaria—an uncommon context for EIA. These findings underscore the role of exercise as a modifiable cofactor that can lower the reaction threshold and exacerbate allergic responses. In our cohort, comorbidities were present in nearly half of the patients, most commonly asthma and malignancy, which were significantly more common in hospital-onset anaphylaxis. Unlike some studies suggesting that asthma is a risk factor for severe outcomes, we found no association between asthma or other comorbidities and anaphylaxis severity, which is consistent with reports questioning its role [9, 25, 30, 35]. Similarly, no link was observed between asthma and hypoxia, respiratory failure, or Pediatric Intensive Care Unit (PICU) admission, contrary to the findings of Brown et al. (37). These findings differ from those of the Polish Anaphylaxis Registry, which identified comorbidity burden as a predictor of anaphylaxis frequency [11], and from data from the Philippines, where malignancy and other comorbidities were strongly associated with severe outcomes [8].

Overall, these findings emphasize the need for a comprehensive evaluation of both endogenous (e.g., comorbidities) and exogenous (e.g., infections, drugs, exercise) factors in patients with anaphylaxis. In our pediatric cohort, comorbidities and atopy did not independently influence severity, whereas polypharmacy and older age were more frequently associated with severe reactions. Comprehensive assessment enables enhanced patient education, individualized risk profiling, and the development of targeted secondary prevention strategies.

### Strengths and limitations

A key strength of this study is the detailed evaluation of comorbidities and cofactors in pediatric anaphylaxis, including their impact on reaction severity—a relatively understudied subject. Nevertheless, several limitations merit consideration. The retrospective design may have resulted in incomplete or missing data, and follow-up attendance was limited to half of the cohort, potentially constraining confirmatory testing. Etiologic clarification for idiopathic cases is further limited by reimbursement restrictions on molecular diagnostics. This is particularly relevant for reactions to processed foods, where precise ingredients and additives may be unknown, and diagnostic testing for known additives could not be performed for the same reasons. Given that this study was conducted at a single tertiary center in Istanbul, the findings may not be fully generalizable to the broader Turkish pediatric population. Prospective, multicenter studies are warranted to validate and extend these observations.

## Conclusion

This study provides important insights into the characteristics of pediatric anaphylaxis in a tertiary care setting.Medications emerged as the most common trigger in our cohort. This contrasts with most pediatric cohorts, where food remains the predominant trigger, likely reflecting the specific characteristics of our study population, including the inclusion of in-hospital reactions. The notable contribution of processed foods underscores the emerging challenge of hidden allergens, whereas rare healthcare- or environment-related triggers highlight the need for heightened clinical vigilance in atypical cases. In our cohort, food-induced reactions and endogenous cofactors were associated with milder presentations, whereas older age, drug-induced reactions, in-hospital onset, and polypharmacy emerged as significant predictors of severe anaphylaxis. Biphasic reactions were associated with delayed presentation to the emergency department, elevated systemic inflammatory markers, and advanced age. Together, these findings contribute to a better understanding of pediatric anaphylaxis risk profiles and highlight the potential importance of judicious medication practices and individualized observation strategies. These findings are specific to our cohort and cannot be directly generalized to other pediatric populations or healthcare settings, but they may offer valuable insights for future studies. Prospective multicenter studies including larger patient cohorts in varied healthcare settings are warranted to confirm and expand upon these findings.

## Data Availability

Research data are not shared. The datasets generated and/or analyzed during the current study are not publicly available owing to patient confidentiality and institutional data protection policies but are available from the corresponding author upon reasonable request.

## Abbreviations

AAI: Adrenaline autoinjector
AAP: Anaphylaxis action plan
ANC: Absolute neutrophil count
CI: Confidence interval
DIA: Drug-induced anaphylaxis
DTP: Diphtheria‒tetanus‒pertussis (vaccine)
ED: Emergency department
EIA: Exercise-induced anaphylaxis
FIA: Food-induced anaphylaxis
IgE: Immunoglobulin E
IQR: Interquartile range
LTP: Lipid transfer protein
NDIA: Nondrug-induced anaphylaxis
NFIA: Nonfood-induced anaphylaxis
NSAID: Nonsteroidal anti-inflammatory drug
OR: Odds ratio
RR: Relative risk
SD: Standard deviation
SPT: Skin prick test
WAO: World Allergy Organization
WBC: White blood cell
